# Geochip-based analysis of microbial communities in alpine meadow soils in the Qinghai-Tibetan plateau

**DOI:** 10.1186/1471-2180-13-72

**Published:** 2013-03-29

**Authors:** Yuguang Zhang, Zhenmei Lu, Shanshan Liu, Yunfeng Yang, Zhili He, Zuohua Ren, Jizhong Zhou, Diqiang Li

**Affiliations:** 1 Institute of Forestry Ecology, Environment and Protection, and the Key Laboratory of Forest Ecology and Environment of State Forestry Administration, the Chinese Academy of Forestry, Beijing, 100091, China; 2 Institute for Environmental Genomics and Department of Botany and Microbiology, the University of Oklahoma, Norman, OK, 73019, USA; 3 College of Life Science, Zhejiang University, Hangzhou, 310058, P. R. China; 4 Department of Environmental Science and Engineering, Tsinghua University, Beijing, 100084, China; 5 Hunan Agricultural University, Changsha, 410128, China

**Keywords:** Alpine meadow, Geochip 3.0, Microbial functional gene diversity, Environmental variable, Climate change

## Abstract

**Background:**

GeoChip 3.0, a microbial functional gene array, containing ~28,000 oligonucleotide probes and targeting ~57,000 sequences from 292 functional gene families, provided a powerful tool for researching microbial community structure in natural environments. The alpine meadow is a dominant plant community in the Qinghai-Tibetan plateau, hence it is important to profile the unique geographical flora and assess the response of the microbial communities to environmental variables. In this study, Geochip 3.0 was employed to understand the microbial functional gene diversity and structure, and metabolic potential and the major environmental factors in shaping microbial communities structure of alpine meadow soil in Qinghai-Tibetan Plateau.

**Results:**

A total of 6143 microbial functional genes involved in carbon degradation, carbon fixation, methane oxidation and production, nitrogen cycling, phosphorus utilization, sulphur cycling, organic remediation, metal resistance, energy process and other category were detected in six soil samples and high diversity was observed. Interestingly, most of the detected genes associated with carbon degradation were derived from cultivated organisms. To identify major environmental factors in shaping microbial communities, Mantel test and CCA Statistical analyses were performed. The results indicated that altitude, C/N, pH and soil organic carbon were significantly (P < 0.05) correlated with the microbial functional structure and a total of 80.97% of the variation was significantly explained by altitude, C/N and pH. The C/N contributed 38.2% to microbial functional gene variation, which is in accordance with the hierarchical clustering of overall microbial functional genes.

**Conclusions:**

High overall functional genes and phylogenetic diversity of the alpine meadow soil microbial communities existed in the Qinghai-Tibetan Plateau. Most of the genes involved in carbon degradation were derived from characterized microbial groups. Microbial composition and structures variation were significantly impacted by local environmental conditions, and soil C/N is the most important factor to impact the microbial structure in alpine meadow in Qinghai-Tibetan plateau.

## Background

Microorganisms are the most abundant and diverse groups of organisms known on our planet, which play key roles in ecosystems and biogeochemical cycling of carbon, nitrogen, sulfur, phosphorus, and metals and biodegradation or stabilization of environmental contaminants [1-3]. Therefore, understanding microbial community structure, diversity, function and their relationships with environmental factors and ecosystem functioning is essential for the research of community formation and sustainability of life on our planet, which facilitates the management and protection of our natural environments [3,4]. Numerous studies have been conducted to investigate the microbial community structure, diversity and their relationships with environments. Some studies showed that the microbial community is very sensitive to environmental changes, compared to plants and animals [5-8]. However, understanding is still limited on soil microbial communities in terms of structure, composition, and functional activity and their impact and response on environmental variations, especial for some special environments.

A large number of molecular approaches were developed and applied to analyze microbial diversity in the last two decades. Among them, high-throughput genomics technologies have shown great potential to study microbial diversity and the driving forces of different ecosystem processes as well as their response to different geological locations and environment changes [8-10]. GeoChip contains probes corresponding to genes encoding key enzymes involved in various biogeochemical cycling, thus it provided rapid, specific, sensitive and potentially quantitative analysis for microbial communities and was useful for studying the functional diversity and dynamics of microbial communities in different natural environments [8,11-14]. Geochip 3.0, containing ~28,000 oligonucleotide probes and targeting ~57,000 sequences from 292 functional gene families, has been used to analyze microbial communities from different habitats of soils [14,15], water [16], oil fields [17], marine sediments [10] and contaminated sites [10-19]. These studies showed that this Geochip served as a powerful tool for researching microbial community structure in natural environments [3].

The Qinghai-Tibetan Plateau, which extends over 2.5 million km^2^, is the youngest, highest and largest geo-morphological unit on the Eurasian continent [20], and was considered “The third pole of Earth”. However, this area also is a key region very sensitive to the impact of global warming. Therefore, the Qinghai-Tibetan Plateau has important significant values in scientific researches [21]. The alpine meadow ecosystem, covering about 35% of the plateau area, is the dominant plant community type of the Qinghai-Tibetan Plateau [22]. *Kobresia*, as one of the dominant genera of alpine meadows, is a typical vegetation on the Qinghai-Tibetan Plateau [23]. At present, some studies found that the majority and diversity functional genes involved in nitrogen fixation and denitrifying existed in the alpine meadow in Qinghai-Tibetan plateau, and altitude and C/N ratio are the important environmental parameters affecting the activity of soil bacteria [20,21]. However, little is known about the functional diversity and metabolic potential at the community level in the alpine meadow, especially for the *Kobreasia*, and the relationship between the functional gene structure of microbial communities and the surrounding environmental factors remains unclear [24].

In this study, Geochip 3.0 was employed to address two key questions. (i) what are the microbial functional gene diversity and structure, and metabolic potential of alpine meadow soil in Qinghai-Tibetan Plateau? (ii) what are the major environmental factors in shaping microbial communities structure in alpine meadow? To answer these questions, six soil samples were obtained and analyzed from the alpine meadow in the center part of the Qinghai-Tibetan Plateau, China.

## Methods

### Site description, sample collection, and geochemical analysis

The study sites were located in Sanjiangyuan Nature Reserve (89°24^′^-102°23^′^E, 31°39^′^-36°16^′^N), in the center of the Qinghai-Tibetan Plateau, China. *Kobresia*, as one of the dominant genera of alpine meadows, is a typical vegetation on the Qinghai-Tibetan Plateau. Six sites of typical *Kobresia* vegetation were selected in this study (Table 1). At each site, three 2 m × 2 m plots comprising typical vegetation were set up and the distance between nearly plots was about 20 m. Five to eight soil cores from the upper layer (0-15 cm) at a diameter of 1.5 cm were collected and mixed equally at each plot, and three plots were mixed and formed a soil sample at each site. Soil samples were stored at -20^o^C.

**Table 1:** Location and geochemistry characteristics of the studied soil samples

**Sample No.**	**Sample location**	**Elevation**	**SOC (g/kg)**	**TN**	**C/N**	**P**	**K**	**AP**	**AK**	**pH-H**_**2**_**O**
				**(g/kg)**		**(g/kg)**	**(g/kg)**	**(mg/kg)**	**(mg/kg)**	
SJY-GH	35°52.524^′^N, 99°56.758^′^E	3400 m	91.99	1.50	61.33	0.59	5.93	14.60	0.80	7.57
SJY-DR	33°34.586^′^N, 99°53.899^′^E	4077 m	93.74	3.10	30.24	0.62	6.15	33.50	0.90	6.09
SJY-QML	34°03.924^′^N, 95°49.240^′^E	4126 m	103.99	4.30	24.18	0.69	6.97	26.20	1.00	7.63
SJY-CD	33°38.200^′^N, 97°11.236^′^E	4412 m	146.25	7.90	18.51	1.28	8.63	40.70	2.10	6.65
SJY-ZD	33°18.194^′^N, 96°17.266^′^E	4457 m	107.06	4.90	21.85	0.75	7.78	40.40	2.20	6.72
SJY-YS	33°21.117^′^N, 96°14.802^′^E	4813 m	209.19	15.50	13.51	1.53	11.92	50.80	1.30	6.73

*SOC* total organic carbon, *TN* total nitrogen, *C/N* total organic carbon to total nitrogen ratio, *P* total phosphorus, *K* total potassium, *AP* available potassium, *AK* available phosphorus.

Soil samples were air-dried, sieved < 2 mm and analysed for pH (1:2 soil to H_2_O ratio), total organic carbon, total nitrogen, total phosphorus, total potassium, available potassium, available phosphorus as previously described [25].

### Soil DNA extraction, purification and labeling

Microbial community genomic DNA was extracted directly from a 5 g soil sample by using a protocol that included liquid nitrogen grinding, freezing and thawing, and treatment with sodium dodecyl sulfate for cell lysis, which has been previously described [26]. Then DNA was purified twice using 0.5% low melting point agarose gel followed by phenol-chloroform-butanol extraction. Purified DNA was quantified with an ND-1000 spectrophotometer (Nanodrop Inc.) and Quant-It PicoGreen (invitrogen, Carlsbd, CA). 3 μg of amplified DNA was labeled with a Cy5 fluorescent dye (GE Healthcare) by a random priming method [12].

### DNA microarray hybridization, scanning and data processing

GeoChip 3.0 was used for DNA hybridization and this Geochip contains DNA probes targeting a total of 57,000 genes involved in key microbial processes [14]. All hybridizations were carried out at 45°C for 10 h with 50% formamide using a TECAN HS4800. Arrays were scanned by using the ScanArray 5000 analysis system (Perkin-Elmer, Wellesley, MA). Signal intensities of each spot were measured with ImaGene 6.0 (Biodiscovery Inc., EI Segundo, CA, USA) and only the spots automatically scored as positive in the output of raw data were used for further data analysis [17]. Spots with a signal-to-noise ratio [SNR = (signal intensity-background intensity)/standard deviation of the background] greater than 2.0 were used for further analysis.

### Statistical analysis

Functional gene diversity was calculated by using Simpson’s reciprocal index (1/D) and Shannon-Weaver index (H’) using online software (http://www2.biology. ualberta.ca/jbrzusto/krebswin/html). Hierarchical clustering analysis of whole functional genes was performed using by the unweighted pairwise average-linkage clustering algorithm with CLUSTER (http://rana.lbl.gov/EisenSoftware.htm) and visualized by TREEVIEW software [27]. The mantel tests were performed using R 2.9.1 (http://www.r-project.org/). Canonical correspondence analysis (CCA) is a multivariate ordination method and was performed to analysis the relationship between microbial community and environment variables by using the program package Canoco for Windows 4.5 (Biometris, The Nerherlands).

## Results

### Geochemical properties in sampling sites

Soil characteristics of these six sampling sites are summarized in Table 1. pH in all those sites was neutral or close to alkali, and they were rich in organic carbon (C) and nitrogen (N), ranging from 91.99 g/kg to 209.19 g/kg and 1.50 g/kg to 15.50 g/kg, respectively. It was noted that C/N ratios displayed a decreasing trend as the elevation increased. For example, sample SJY-GH with the lowest elevation (3400 m) had the highest value of C/N ratio, whereas sample SJY-YS with the highest elevation (4813 m) had the lowest C/N ratio. In addition, sample SJY-GH had higher total C, N, P and K contents than the other samples.

### Overview of functional gene diversity and structure of soil microbial communities

The examined microbial communities showed high diversity, as judged by the number of detected genes, overlapping genes between samples, unique genes and diversity indices (Table 2). The total number of detected genes ranged from 1,732 to 3,746 among the six study sites (Table 2). For instance, twice as many genes were detected in sample SJY-GH as in sample SJY-CD, SJY-ZD or SJY-YS. These samples had different community compositions, as shown by the unique and overlapped genes (Table 2). Sample SJY-GH and sample SJY-DR had the most overlapped genes (2029, 42.94%), while sample SJY-GH and sample SJY-YS had the fewest overlapped genes (1178, 27.22%). Simpson’s reciprocal diversity index (1/D) was the highest in sample SJY-GH and the lowest in sample SJY-CD (3716 and 1723, respectively). Similar results were obtained with Shannon-Weaver index (Table 2).

**Table 2:** **Total detected gene number, gene overlap, unique, diversity indices of soil sample**^**a**^

**Unique and overlap genes**	**SJY-GH**	**SJY-DR**	**SJY-QML**	**SJY-CD**	**SJY-ZD**	**SJY-YS**
SJY-GH	**1044(27.87%)**	2029(42.94%)	1655(37.26%)	1264(30.00%)	1261(29.84%)	1178(27.22%)
SJY-DR		**617(20.51%)**	1485(38.33%)	1171(32.81%)	1163(32.43%)	1107(30.24%)
SJY-QML			**403(17.14%)**	1049(34.57%)	1062(35.05%)	973(31.01%)
SJY-CD				**242(13.97%)**	916(35.82%)	840(31.67%)
SJY-ZD					**248(14.24%)**	816(30.39%)
SJY-YS						**321(18.24%)**
**Total no. of genes detected**	3746	3008	2351	1732	1741	1760
**Shannon weaver index**	8.22	8.01	7.76	7.45	7.46	7.47
**Simpson’s reciprocal diversity index (1/D)**	3716	2988	2340	1723	1733	1752

^a^ Values in parentheses are percentages. Italicized values indicate the number of overlapping genes between samples, boldface values indicate the number of unique genes in each sample.

According to the phylogenetic analysis, the *Proteobacteria* group is the most dominant bacteria in all six samples, which account for over 56% (over 23% belong to α-proteobacteria, 13% belong to β-proteobacteria, 14% belong to γ-portecobacteria) among all the detected genes (Additional file 1: Table S1). The Actinobacteria (over 9.30%) and firmicutes (3.73%) are the second and third dominant bacteria groups. The genes derived from the Archaea and Eukaryota also were detected and accounted for 1.64% to 2.04% and 4.35% to 5.33% among all the detected genes in all samples, respectively. Although gene numbers belonging to different phylogenetic structure varied considerably in different samples, the proportions of genes number of different phylogenetic structure in all detected genes is similar. For example, the ratio of *α-Proteobacteria* ranged from 23.18% to 24.99% and the ratio of *Actinobacteria* ranged from 9.30% to 10.97% (Additional file 1: Table S1). Therefore, these results indicated the overall functional genes as well as the phylogenetic diversity of these alpine meadow soil microbial communities appeared to be quite high.

### Analysis of detected functional genes

Among the 6143 genes detected in at least one sample, 567 were involved in carbon degradation, 202 in carbon fixation, 36 in methane oxidation, 18 in methane production, 754 in nitrogen cycling, 153 in phosphorus utilization, 279 in sulphur cycling, 2540 in organic remediation, 1275 in metal resistance, 126 in energy process, 193 in other category. Detected functional genes among these six alpine meadow soil samples were analyzed by hierarchical clustering (Additional file 1: Figure S1). A total of 39 different clusters of genes were observed. Genes in group 5, group 32 and group 35 are presented in all of the samples. The most obvious patterns were group 11 (1054 [17.16%]) and group 33 (373 [6.07%]); instead of, the genes in group 11 is only present at sample SJY-GH which is the lowest altitude sample and group 33 is only present at sample SJY-YS which is the highest altitude sample. The genes in group 11 were from functional categories involved in carbon degradation, carbon fixation, denitrification, nitrification, nitrogen fixation, phosphorus utilization, sulfite reductase, etc. Most of the genes in group 33 are involved in the carbon degradation, denitrification, nitrogen fixation, organic remediation, etc. These results showed that different microbial community structures existed in these samples and environment factors may influence them. To better understand microbial diversity involved in soil carbon cycling and nitrogen cycling, selected gene groups were further analyzed.

### Functional genes involved in the carbon cycling

Microbe-mediated carbon cycling is one of the most important and complex process in the biogeochemical cycling. A total of 5196 gene probes belonging to carbon cycling were detected in the Geochip 3.0 [14]. Among them, 823 gene probes were detected in all six soil samples (Table 3). Sample SJY-GH and SJY-CD have the most and least detected gene numbers, respectively. Carbon fixation and carbon degradation are the two most important gene categories in the carbon cycling in all samples (Table 3). The key enzymes (CODH, FTHFS, pcc and rubisco) involved in carbon fixation were detected in all samples and most of the detected genes (198/202) are derived from cultured bacteria. For example, fourteen genes were derived from *Rhodopseudomonas palustris*, four genes were derived from *Xanthobacter autotrophicus*, four genes were derived from *Verminephrobacter eiseniae*, three genes were derived from *Roseiflexus Sp.* and two genes were derived from *Burkholderia xenovorans*. However, only a few number of genes (10/202) involved in carbon fixation were shared by all six samples and *Roseiflexus Sp.* and *Burkholderia xenovorans* have high signal intensity in all of these soil samples.

**Table 3:** The detected gene probes number involving in carbon and nitrogen cycling

**Gene category**	**Detected No. of probes**	**Detected gene probes number in different sampling sites**					
		**SJY-GH**	**SJY-DR**	**SJY-QML**	**SJY-CD**	**SJY-ZD**	**SJY-YS**
**Carbon cycling**	**823**	**466**	**359**	**300**	**207**	**232**	**228**
Carbon fixation	202	108	81	83	52	54	46
Carbon degradation	567	336	252	196	145	160	162
Strarch	161	91	66	54	39	45	43
Cellulose	63	41	24	23	16	14	22
Hemicellulose	105	61	55	38	27	27	27
Lignin	76	53	37	31	23	22	23
Chitin	90	49	36	24	20	34	23
Pectin	12	7	6	5	0	2	3
Others	60	34	28	21	20	16	21
Methane production	18	6	6	5	3	8	5
Methane oxidation	36	16	20	16	7	10	15
**Nitrogen cycling**	**754**	**433**	**366**	**287**	**195**	**206**	**199**
Nitrogen fixation	224	116	108	79	52	56	62
Denitrification	372	222	185	143	97	100	96
Nitrification	17	7	8	4	3	4	2
Dissimilatory N reduction	51	34	24	18	12	20	15
Assimilatory N reduction	27	11	7	14	8	7	9
Anaerobic ammonium oxidation	63	43	34	29	23	19	15

Genes involved in the degradation of starch, cellulose, hemicellulose, chitin, lignin and pectin also were detected in Geochip and 161, 63, 105, 76, 90 and 12 gene probes were detected in all six samples (Table 3). All of the detected genes involved in the degradation of starch, cellulose and hemicellulose were derived from the cultured bacteria, and over 80% detected genes involved chitin, lignin and pectin (72/76, 85/90 and 10/12, respectively) were derived from cultured bacteria. However, only a few genes involved in the degradation of starch, cellulose, hemicellulose, chitin, lignin and pectin (14/161, 5/63, 6/105, 8/76, 8/90 and 0/12, respectively) were shared by all six samples. For methane cycle, a higher gene number and signal intensity of methane oxidation genes (*mmoX* and *pmoA*) were detected than that of methane production genes (*mcrA*) in all six samples. Most of the genes involved in methane oxidation and production (32/36 and 16/18) are derived from the uncultured microorganisms.

Most of shared genes involved in carbon cycling have high signal intensity in all the samples. For example, cellobiase gene involved in cellulose degradation derived from *Roseiflexus castenholzii* DSM 13941 was abundant in and shared by all six samples (Additional file 1: Figure S2), and gene derived from *Rhodococcus* sp. RHA1, *Trichoderma harzianum* and *Arthrobacter* sp. FB24 were also abundant. These results indicated that all of the processes involved in carbon cycling existed in the alpine meadow, and there were abundant genes diversity and most of the genes derived from the cultured bacteria in the Qinghai-Tibetan Plateau.

### Functional genes involved in the nitrogen cycling

A total of 3763 gene probes belonging to different key gene categories involved in nitrogen fixation, denitrification, nitrification, dissimilatory N reduction, assimilatory N reduction and anaerobic ammonium oxidation are present in Geochip 3.0 [14]. Among them, 754 gene probes were detected in all six soil samples (Table 3). 224, 372, 17, 51, 27 and 63 genes involved in nitrogen fixation, denitrification, nitrification, dissimilatory N reduction, assimilatory N reduction and anaerobic ammonium oxidation were detected in all samples, respectively (Table 3). Sample SJY-GH and SJY-CD have the most and least detected gene number, respectively.

Microbe-mediated nitrogen fixation and denitrification are the most important processes in nitrogen cycling. Microbe-mediated nitrogen fixation is the most important source of nitrogen in natural ecosystems, and occurs across a wide range of bacteria phyla, from Archaebacteria to Eubacteria [28]. The majority of *nifH* genes (155/224) were derived from unidentified or uncultured organisms retrieved from different environments. Among *nifH* genes, 19 were shared by all samples. The shared gene 44829093 derived from an uncultured bacterium was dominant in samples SJY-GH and SJY-YS, and 780709 from an unidentified marine eubacterium was the most dominant gene in sample SJY-CD. These samples had a relatively high abundance of genes involved in nitrogen fixation.

Denitrification is a dissimilatory process of denitrifying bacteria where oxidized nitrogen compounds are used as alternative electron acceptors and nitrogen is transferred into the atmosphere in form of N_2_. Most of the detected genes involved in denitrification (320/372) were derived from the unidentified or uncultured organisms retrieved from different environments. These samples had a relatively high abundance of genes involved in denitrification (Table 3). 67 *nosZ* genes which encoding nitrous oxide reductase and it is considered a key enzyme in the denitrification process were detected. Few genes (13/67) were derived from the isolated bacteria. Four genes were shared and derived from the uncultured bacteria by all six soil samples (Additional file 1: Figure S3).

Together, these results indicated that all the processes involved in nitrogen cycling existed, and there were high gene diversity as well as high potential metabolic ability in nitrogen fixation and denitrification in all these samples.

### Relationships between microbial community structure and environmental variables

**Figure 1:**
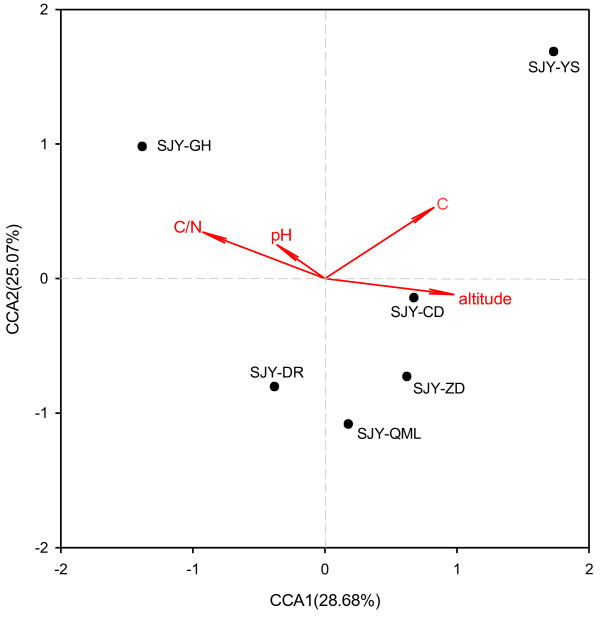
Canonical correspondence analysis (CCA) of Geochip hybridization signal intensities and soil environmental vairables significantly related to microbial community variations: altitude (A), the ratio of organic carbon and total nitrogen (C/N), pH and Soil organic carbon (C).

To assess the relationships between microbial community structure and soil environmental variables, Mantel test and canonical correspondence analysis (CCA) were used. Mantel tests of all six soil samples were performed with 12 individual environmental variables. The environmental variables of altitude, C/N, pH and soil organic carbon were used to analyze the correlation with the microbial functional genes involved in carbon and nitrogen cycling (Additional file 1: Table S2), suggesting that these environmental variables play important roles in shaping the microbial community structure in these soil samples. Consistently, CCA results showed that the C/N and altitude were the most important factors when only significant environmental variables (altitude, C/N, pH and organic carbon) were included in the CCA biplot (Figure 1). Samples of SJY-DR, SJY-CD, SJY-ZD and SJY-QML clustered together which were separated from in SJY-GH and SJY-YS (Figure 1). On the basis of the relationship between environmental variables and microbial functional structure, altitude seemed to be the most important variable affecting the microbial functional structure. Notably, sample SJY-GH was collected at a low altitude (3400 m), while sample SJY-YS was collected at a high altitude (4813 m), while the altitude of Sample SJY-DR, SJY-CD, SJY-ZD and SJY-QML was 4000-4500 m.

**Figure 2:**
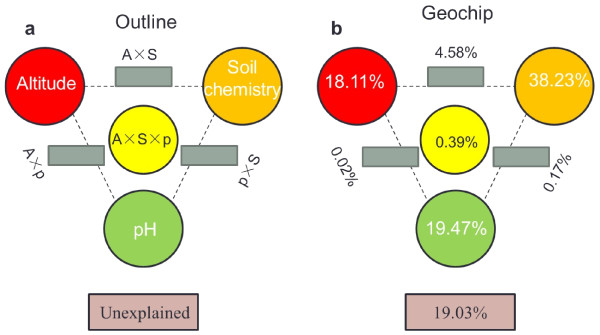
**Variation partitioning analysis of microbial diversity explained by sample altitude (A), soil geochemistry factors (S) and pH (p).** (**a**) General outline, (**b**) all functional genes. Each diagram represents the biological variation partitioned into the relative effects of each factor or a combination of factors, in which geometric areas were proportional to the respenctive percentages of explained variation. The edges of the triangle presented the variation explained by each factor alone. The sides of the triangels presented interactions of any two factors, and the middle of the triangles represented interactions of all three factors.

Variance partitioning analysis was used to quantify the contributions of altitude (A), soil chemistry (S) and pH (p) to the microbial community variation. The total variation was partitioned into the independent effects of A, S and pH (when the effects of all other factors were removed), interactions between only two factors, common interactions of all three factors and the unexplained portion (Figure 2a). On the basis of Geochip data, a total of 80.97% of the variation was significantly explained by these three environmental variables (Figure 2b). Altitude, C/N and pH were able to independently explain 18.11%, 38.23% and 19.47% of the total variations observed, respectively. Interactions between any two factors or among the three factors seemed to have less effect than the individual factors. Only about 20% of the community variation could not be explained by these three environmental variables.

## Discussion

Analyzing microbial functional genes involved in major biogeochemical processes is important to link microbial community structure to their potential ecological functions [29]. In this study, we used GeoChip 3.0 to analyze microbial functional gene diversity in alpine meadow soil samples from the Qinghai-Tibetan plateau. This report was one of the first ecological applications of an expanded functional gene microarray [13,30], and it is the first application of this kind for studies in Qinghai-Tibetan plateau, China. These results indicated the overall functional genes as well as the phylogenetic diversity of these alpine meadow soil microbial communities is higher than in the Antarctic latitudinal transect or alpine soil in the Colorado Rocky Mountains [30,31]. All the detected genes involved in the carbon degradation, carbon fixation, methane oxidation and production, nitrogen cycling, phosphorus utilization, sulphur cycling, organic remediation, metal resistance, energy process, and other category. According to the phylogenetic analysis, the proteobacteria group is the most dominant bacteria in all six samples, which account for over 56% among all the detected genes. Therefore, *Proteobacteria* maybe the most prevalent bacteria in Qinghai-Tibetan plateau.

Soil is the major reservoir of terrestrial organic carbon, and soil carbon degradation is largely controlled by the metabolic activities of the microorganisms present in the soil [32,33]. The majority of microbial studies have monitored the relationship between organic carbon in soil, CO_2_ release, and microbial biomass in different soil types [34,35]. In this study, metabolic genes involved in the degradation of starch, cellulose, hemicellulose, chitin, lignin and pectin were detected and the individual gene orthologs were abundant and diverse. For example, 76 genes related to lignin degradation were detected and the number of genes detected was 53, 37, 31, 23, 22 and 23 in SJY-GH, SJY-DR, SJY-QML, SJY-CD, SJY-ZD and SJY-YS, respectively. These detected genes related to lignin degradation belonged to 4 different gene families, including laccase, glyoxal oxidase, lignin peroxidase and manganese peroxidase, and most of the detected genes (94.59%) were derived from the isolated organisms (e.g., 17.57% from *Phanerochaete* sp.). Most of the shared genes were abundant in all the samples. For example, the cellobiase gene involved in cellulose degradation derived from *Roseiflexus castenholzii* DSM 13941 was shared by all of the six samples and had the highest signal intensity in all samples.

Understanding the environmental variables that affect microbial community structure is a key goal in microbial ecology [17]. Different environmental variables affect the microbial structure and potential activity on ecosystem functions [15]. He et al [15] found that the abundance of all detected genes was significantly (P < 0.05) and positively correlated with soil moisture and pH. Yergeau et al. [30] used the Geochip to examine soil microbial communities across an Antarctic latitudinal transect and revealed that cellulose degradation and denitrification genes were correlated with soil temperature. Our results showed that altitude, C/N, pH and available phosphorus had a significant impact on the microbial functional communities in alpine meadow soil, suggesting that these environmental variables play an important role in shaping microbial community structure. However, we know very little about how microbial distribution pattern varies along altitude gradients [36]. This is a considerable gap in understanding microbial biodiversity and will likely be an important component of ecosystem response to global warming [37,38].

Variation partitioning analysis in this study showed that a total of 80.97% of the variation was significantly explained by altitude, C/N and pH. The C/N contributed the most (38.2%) to microbial functional gene variation, which is in accordance with the hierarchical clustering of overall microbial functional genes, indicating a significant impact of local environmental conditions on the composition and structures of microbial communities. In this study, only 19.03% of the variation of microbial community structure could not be explained by of these three factors, which showed that considerable amounts of variations could be explained by environmental variables measured. However, some previous studies thought that most of the variation could be explained by environmental variables. For example, Zhou et al. [8] showed that more than 50% of variations in a forest soil community could not be explained by both environmental factors and geographic distance. Ramette and Tiedje [39] showed that 34-80% of microbial variations could not be explained by measured environmental variables in agricultural soils. Liang et al [17] indicated over 40% of the variations of microbial community could not be explained by geographic location, soil geochemical variables and oil contamination.

In summary, soil microbial functional gene diversity in alpine meadow in Qinghai-Tibetan plateau was examined by Geochip 3.0 and almost all genes involved in carbon, nitrogen and other element cycling were found, which showed that the microbial functional diversity in alpine meadow ecosystem was quietly high. Statistical analyses showed that the microbial communities may be shaped largely by the altitude, C/N, and pH. However, Geochip analyzed the distribution of metabolic genes may reflect the metabolic potential of the microbial community [27], but not necessarily the actual populations. For example, we detected many key enzyme genes involved in carbon degradation, which implied that the populations carrying those genes could exist in the alpine meadow ecosystem, but it does not mean that they express the enzymes of degradation organic carbon. Therefore, further analysis of the functional activity with different approaches such as mRNA-based microarray hybridization is needed to address it [27].

## Conclusions

A highly overall functional genes and phylogenetic diversity of the alpine meadow soil microbial communities existed in the Qinghai-Tibetan Plateau. Most of the genes involved in carbon degradation were derived from characterized microbial groups. The considerable amounts of microbial the composition and structures variation was significant impacted by local environmental conditions, and the C/N is the most important factors to impact the microbial structure in alpine meadow in Qinghai-Tibetan plateau.

## Availability of supporting data

The data set supporting the results of this article is available in the microarray data repository, unique persistent identifier and hyperlink to dataset(s) in http://ieg2.ou.edu/NimbleGen/analysis.cgi

## Additional file

## Competing interests

We declared that this manuscript have not any finical competing interests. We have not received reimbursements, fees, funding, or salary, or hold any stocks or shares from any organizations that may in any way gain or lose financially from the publication of this manuscript, either now or in the future. We also have not hold or apply any patents relating to content of the manuscript. No other financial competing interests are related to this manuscript. We declared that this manuscript have not any non-financial competing interests (political, personal, religious, ideological, academic, intellectual, commercial or any other).

## Authors’ contributions

Y Z carried out the lab design, sampling collecting, data analysis and the manuscript preparation. Z L carried out the soil microbial DNA extraction, microarray hybridization, scanning and data processing. S L participated the microarray data analysis. Y Y participated the microarray data analysis and manuscript preparation. Z R participated the sampling collecting and biogeochemical data analysis. J Z participated the lab design and data analysis. D L participated the lab design, data analysis and manuscript preparation. All authors read and approved the final manuscript.

## Supplementary Material

Additional file 1: Table S1 Distribution of detected genes’ phylogenetic structure in all six soil samples from Qinghai-Tibetan Plateau, China. **Table S2**. The relationship of microbial functional genes involved in carbon and nitrogen cycling to individual environmental variables revealed by Mantel test. **Figure S1**. The hierarchical cluster of the six soil samples based on the signal intensity of all detected genes. The figure was generated by CLUSTER and visualized by TREEVIEW. Black represents no hybridization above background levels, and red represents positive hybridization. The color intensity indicates differences in hybridization signal. Average signal intensities of these groups for each sample are shown on the right. **Figure S2**. The hierarchical cluster analysis of community relationships of cellobiase genes based on hybridization signals for all five soil samples in Qinghai-Tibetan Plateau. The figure was generated by using CLUSTER and visualized with TREEVIEW. Black represents no hybridization above background level, and red represents positive hybridization. The color intensity indicates differences in hybridization patterns. **Figure S3.** The hierarchical cluster analysis of community relationships of *nosZ* genes based on hybridization signals for all five soil samples in Qinghai-Tibetan Plateau. Click here for file
