# Supplementary material for: Geochip-based analysis of microbial communities in alpine meadow soils in the Qinghai-Tibetan plateau
Source: BMC Microbiol. 2013 Mar 29;13:72. doi: 10.1186/1471-2180-13-72 (PMC3617080; doi:10.1186/1471-2180-13-72)
Supplement: Additional file 1: Table S1 — Distribution of detected genes’ phylogenetic structure in all six soil samples from Qinghai-Tibetan Plateau, China. Table S2. The relationship of microbial functional genes involved in carbon and nitrogen cycling to individual environmental variables revealed by Mantel test. Figure S1. The hierarchical cluster of the six soil samples based on the signal intensity of all detected genes. The figure was generated by CLUSTER and visualized by TREEVIEW. Black represents no hybridization above background levels, and red represents positive hybridization. The color intensity indicates differences in hybridization signal. Average signal intensities of these groups for each sample are shown on the right. Figure S2. The hierarchical cluster analysis of community relationships of cellobiase genes based on hybridization signals for all five soil samples in Qinghai-Tibetan Plateau. The figure was generated by using CLUSTER and visualized with TREEVIEW. Black represents no hybridization above background level, and red represents positive hybridization. The color intensity indicates differences in hybridization patterns. Figure S3. The hierarchical cluster analysis of community relationships of nosZ genes based on hybridization signals for all five soil samples in Qinghai-Tibetan Plateau. [file 1471-2180-13-72-S1.doc]

**Supplementary Information**

Table S1. Distribution of detected genes’ phylogenetic structure in all six soil samples from Qinghai-Tibetan Plateau, China

|  | Sample, no. (%) | | | | | |
| --- | --- | --- | --- | --- | --- | --- |
|  | A | B | C | D | E | F |
| Proteobacteria | 2158 (56.89) | 1720 (56.52) | 1368 (57.62) | 1042 (59.58) | 1019 (57.60) | 1044 (58.13) |
| α-proteobacteria | 910 (23.99) | 718 (23.60) | 569 (23.97) | 437 (24.99) | 410 (23.18) | 418 (23.27) |
| β-proteobacteria | 540 (14.24) | 411 (13.51) | 335 (14.11) | 260 (14.87) | 234 (13.23) | 262 (14.59) |
| γ-portecobacteria | 540 (14.24) | 447 (14.69) | 344 (14.49) | 254 (14.52) | 296 (16.72) | 273 (15.20) |
| δ-protecobacteria | 136 (3.59) | 113 (3.71) | 99 (4.17) | 66 (3.77) | 58 (3.28) | 68 (3.79) |
| Actinobacteria | 416 (10.97) | 319 (10.48) | 251 (10.57) | 188 (10.75) | 186 (10.51) | 167 (9.30) |
| Firmicutes | 154 (4.06) | 143 (4.70) | 93 (3.92) | 77 (4.40) | 69 (3.90) | 67 (3.73) |
| Archaea | 69 (1.82) | 57 (1.87) | 39 (1.64) | 29 (1.66) | 36 (2.04) | 31 (1.73) |
| Eukaryota | 202 (5.33) | 137 (4.50) | 113(4.76) | 76 (4.35) | 84 (4.75) | 89 (4.96) |

Table S2. The relationship of microbial functional genes involved in carbon and nitrogen cycling to individual environmental variables revealed by Mantel test

| Environmental variables | Functional genes involved in Carbon cycling | | Functional genes involved in Nitrogen Cycling | |
| --- | --- | --- | --- | --- |
|  | *r_M_* | *P* | *r_M_* | *P* |
| Altitude | 0.8748 | **0.006** | 0.7485 | **0.001** |
| Organic carbon | -0.7116 | 0.971 | -0.6894 | 0.96 |
| Total nitrogen | -0.4167 | 0.95 | -0.4239 | 0.928 |
| Carbon nitrogen ratio | 0.9294 | **0.004** | 0.7528 | **0.006** |
| Total phosphorus | -0.5273 | 0.951 | -0.6042 | 0.984 |
| Total potassium | -0.3943 | 0.893 | -0.2147 | 0.794 |
| Available phosphorus | 0.7621 | **0.026** | 0.6801 | **0.040** |
| Available potassium | 0.1005 | 0.35 | 0.1395 | 0.206 |
| pH | 0.5042 | **0.034** | 0.5861 | **0.038** |


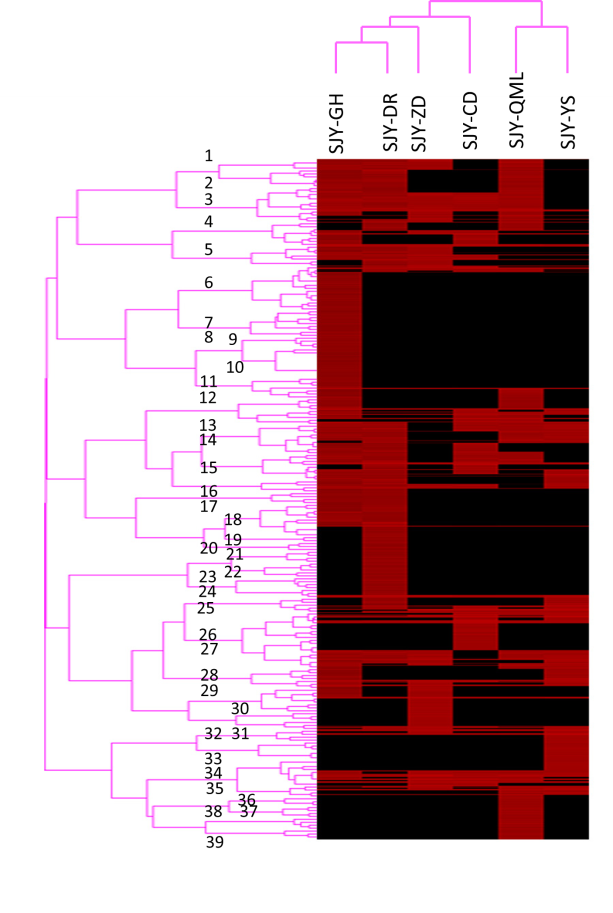


Fig.S1 The hierarchical cluster of the six soil samples based on the signal intensity of all detected genes. The figure was generated by CLUSTER and visualized by TREEVIEW. Black represents no hybridization above background levels, and red represents positive hybridization. The color intensity indicates differences in hybridization signal. Average signal intensities of these groups for each sample are shown on the right.


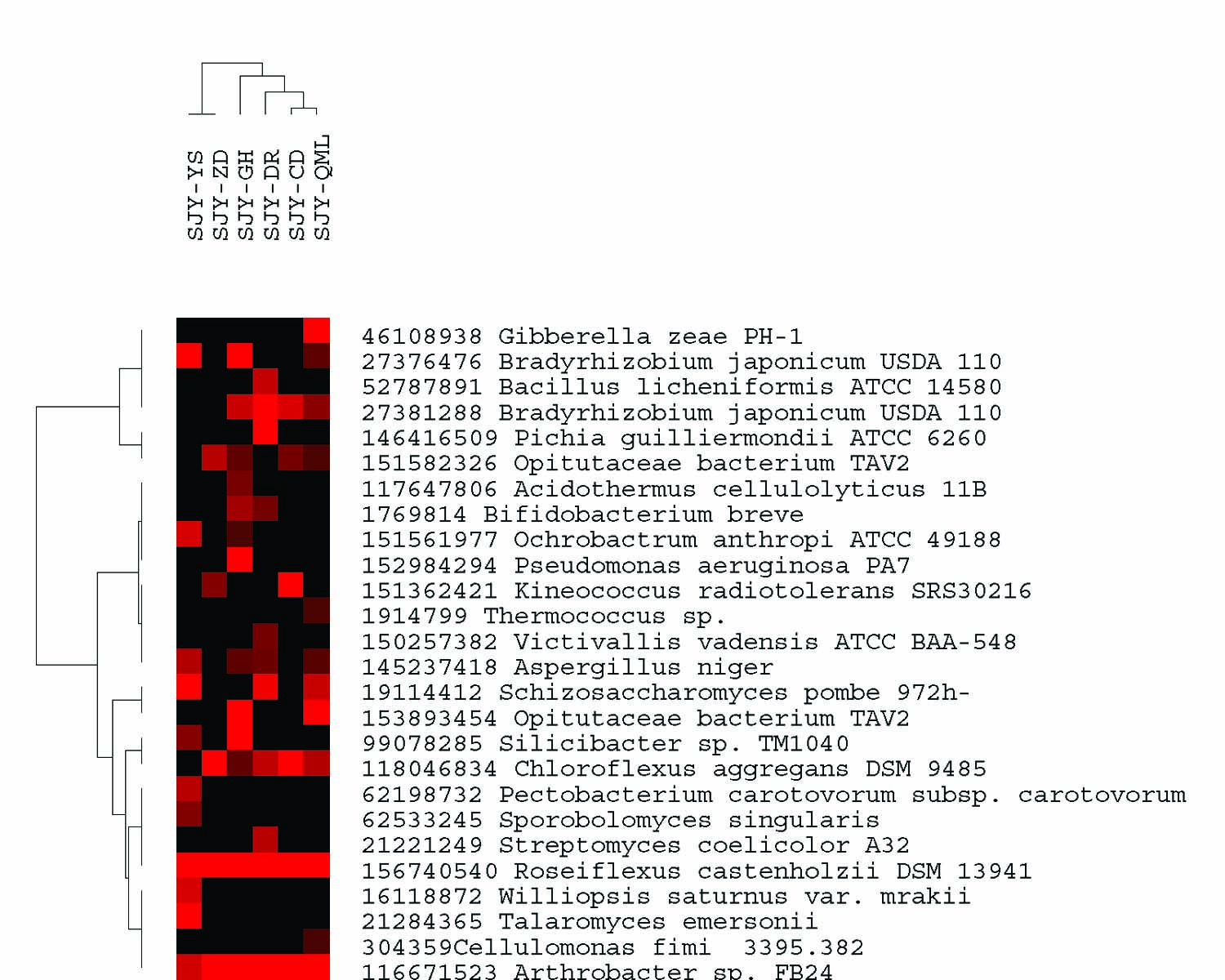


**Fig.S2** The hierarchical cluster analysis of community relationships of cellobiase genes based on hybridization signals for all five soil samples in Qinghai-Tibetan Plateau. The figure was generated by using CLUSTER and visualized with TREEVIEW. Black represents no hybridization above background level, and red represents positive hybridization. The color intensity indicates differences in hybridization patterns.


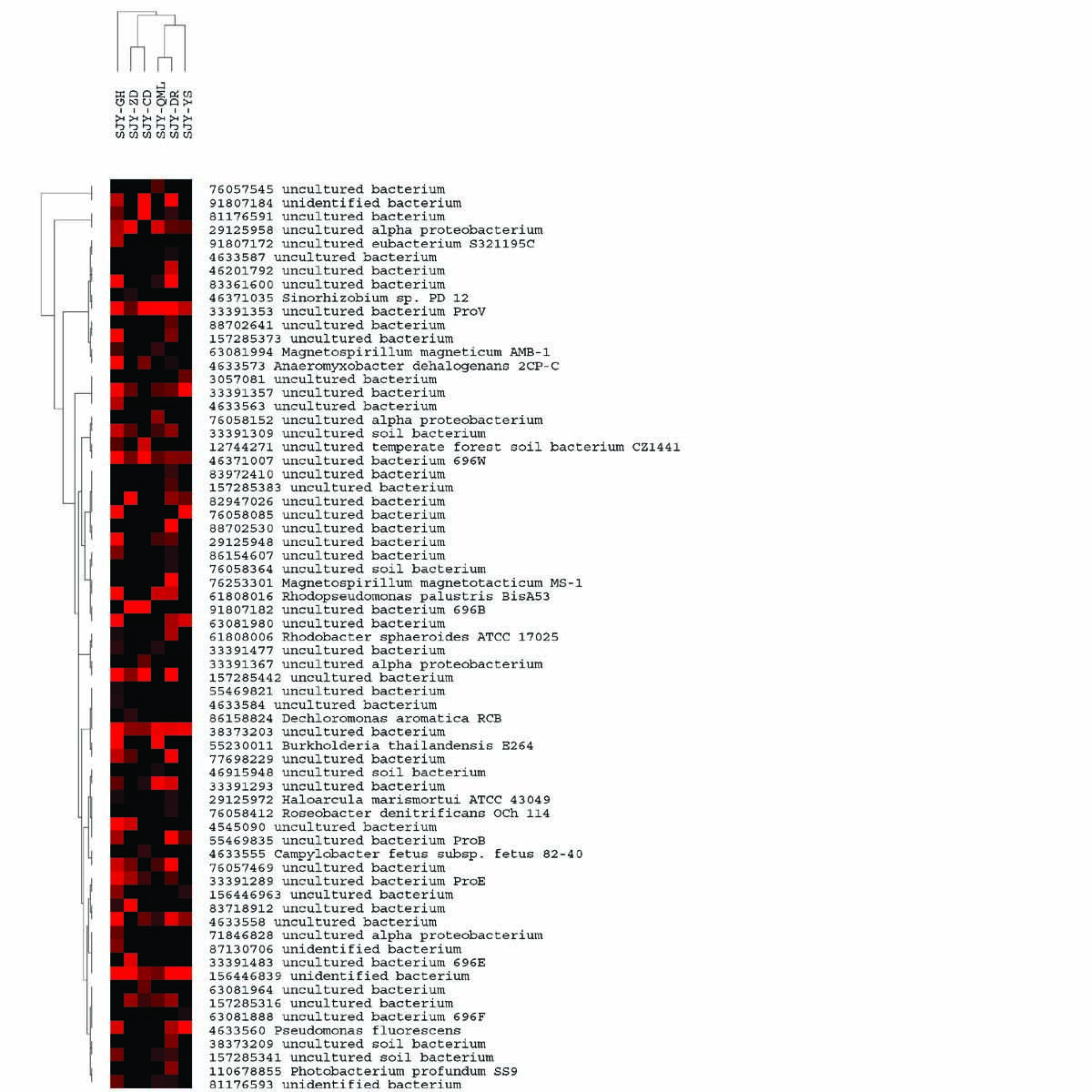


**Fig.S3** The hierarchical cluster analysis of community relationships of *nosZ* genes based on hybridization signals for all five soil samples in Qinghai-Tibetan Plateau.
